# Prognostic markers could extend adjuvant immunotherapy to high-risk stage IB/IIA melanoma: A modeling study

**DOI:** 10.1016/j.jdin.2024.09.010

**Published:** 2024-10-30

**Authors:** Samuel X. Tan, Nicholas Michael Muller, Chenhao Zhou, Euan Walpole, B. Mark Smithers, David C. Whiteman, Kiarash Khosrotehrani

**Affiliations:** a Frazer Institute, Dermatology Research Centre, Experimental Dermatology Group, The University of Queensland, Brisbane, Australia; b Department of Dermatology, Princess Alexandra Hospital, Brisbane, Australia; c Division of Cancer Services, Princess Alexandra Hospital, Brisbane, Australia; d Queensland Melanoma Project, University of Queensland, Princess Alexandra Hospital, Brisbane, Australia; e Department of Population Health, QIMR Berghofer Medical Research Institute, Brisbane, Australia

**Keywords:** adjuvant therapy, biomarkers, melanoma, immunotherapy, precision medicine, prognosis

*To the Editor:* Prescribing adjuvant immunotherapy for melanoma involves balancing the likelihood of benefit against the risk of adverse events. Although the cutoff for treatment has conventionally been set at stage III/IV disease, this threshold is anatomically defined and does not uniformly correlate with prognosis. For example, 5-year melanoma-specific survival (MSS) rates for stage IIB (87%) and IIC (82%) melanoma are comparable to that of stage IIIB melanoma (83%).^1^

Several phase 3 trials have sought to address this disparity by investigating whether adjuvant immunotherapy could be extended to stage IIB/IIC melanoma. In KEYNOTE-716, adjuvant pembrolizumab was associated with higher recurrence-free survival (RFS) (hazard ratio [HR]: 0.64) and distant metastasis-free survival (DMFS) (HR: 0.64) versus placebo in patients with resected stage IIB/IIC melanoma.^2^ The recent interim analysis of CheckMate 76K reported similar improvements in both RFS (HR: 0.42) and DMFS (HR: 0.47) for adjuvant nivolumab versus placebo.^3^

From these trials, we can infer that adjuvant immunotherapy confers similar RFS and DMFS benefits in locally invasive as well as metastatic melanoma. In the context of personalized risk stratification, this establishes a precedent to consider adjuvant therapy for patients with high-risk early-stage disease; for example, those with a predicted 5-year MSS comparable to that of stage IIIB melanoma, or ≤83%.^1^ Here, we modeled whether existing prognostic markers could identify stage IB/IIA melanoma patients who also met this criterion (Supplementary Information, available via Mendeley at https://data.mendeley.com/datasets/9jhm839ck5/1).

Table I presents initial 5-year MSS rates from the American Joint Committee on Cancer Staging Manual, eighth edition.^1^ These are stratified according to relative risk and marker prevalence data from 2 prognostic cohort studies (n: 21,414) of anatomical location and 31-gene expression profile testing in patients with stage I-IIA melanoma.^4^^,^^5^ Under this framework, patients with stage IB scalp melanoma, stage IIA scalp melanoma, or stage IIA/31-gene expression profile class 2B melanoma have estimated 5-year MSS rates of 80.6%, 61.3%, and 67.9%, respectively. Although the predicted survival of stage IB/IIA patients in these 3 prognostic subgroups is lower than that of patients with stage IIIB melanoma, current management guidelines recommend observation alone.

**Table I:** Estimation of 5-year MSS by melanoma stage and prognostic strata

Melanoma stage	Unstratified∗	Scalp melanoma†	31-GEP class 2B‡
Stage IA	99.0%	93.5%	94.7%
Stage IB	97.0%	**80.6%**	84.0%
Stage IIA	94.0%	**61.3%**	**67.9%**
Stage IIIB (comparator)	83.0%		

Modeling was performed using both prevalence and relative risk, as described in the Supplementary Information, available via Mendeley at https://data.mendeley.com/datasets/9jhm839ck5/1.

Bolded values indicate stage-specific subgroups with an estimated 5-year melanoma-specific survival below that of the Stage IIIB comparator.

*GEP*, Gene expression profile; *MSS*, melanoma-specific survival.

∗ Values obtained from Gershenwald et al (AJCC 8).^1^

† Claeson et al (stage I; *n*: 17,376, prevalence_scalp_: 1.09%, 5-year MSS relative risk: 6.96).^4^

‡ Bailey et al (stage I-IIA; *n*: 4038; prevalence_Class 2B_: 7.38%; 3-year MSS relative risk: 8.19).^5^

Adjuvant immunotherapy is not indicated in stage IB/IIA melanoma due to low individual risk and high incidence of immune-related toxicities, many of which induce lifelong morbidity. However, stage IB/IIA melanomas are highly prevalent and account for over one-third of collective melanoma deaths.^4^ We show that published prognostic markers can already identify high-risk patients who disproportionately contribute to this mortality burden yet are overlooked by conventional staging-based schema.

This model is limited by its generalization of single cohorts to wider population data, though both included studies mitigated this through sampling from nationally representative cancer registries. Moreover, overall survival data are yet to mature for KEYNOTE-716 and CheckMate 76K, and may exhibit a smaller effect than for RFS in early-stage melanoma. Lastly, we have only evaluated 2 prognostic studies in this model.

Melanoma risk stratification is an evolving field, and both current and novel prognostic markers require independent validation. Nonetheless, the existing evidence base is sufficient to consider prospective trials of adjuvant immunotherapy in high-risk stage IB/IIA melanoma.

## Conflicts of interest

None disclosed.
